# Physical Activity in Puberty Is Associated with Total Body and Femoral Neck Bone Mineral Characteristics in Males at 18 Years of Age

**DOI:** 10.3390/medicina55050203

**Published:** 2019-05-23

**Authors:** Reeli Tamme, Jaak Jürimäe, Evelin Mäestu, Liina Remmel, Priit Purge, Eva Mengel, Vallo Tillmann

**Affiliations:** 1 Institute of Clinical Medicine, University of Tartu, 50406 Tartu, Estonia; vallo.tillmann@kliinikum.ee; 2 Children’s Clinic of Tartu University Hospital, 50406 Tartu, Estonia; evamengel0@gmail.com; 3 Institute of Sports Sciences and Physiotherapy, University of Tartu, 51007 Tartu, Estonia; jaak.jurimae@ut.ee (J.J.); evelin.maestu@ut.ee (E.M.); liina.remmel@ut.ee (L.R.); priit.purge@ut.ee (P.P.)

**Keywords:** physical activity, puberty, bone mineral density, bone mineral content, adolescence

## Abstract

*Background and objectives:* Studies indicate that genetic and lifestyle factors influence optimal bone development. Adaptations in bone mineral characteristics related to physical activity (PA) are most often observed in pre- and peri-puberty. Longitudinal associations between bone mineral accrual and objectively measured PA in puberty are poorly understood. The present study aims to investigate whether pubertal PA at different intensities is related to bone mineral characteristics in individuals at 18 years of age. *Materials and Methods:* Anthropometrics, pubertal stage, bone age and PA by accelerometer were measured in 88 boys at the mean age of 12.1 (T1), 13.1 (T2), 14.0 (T3) and 18.0 years (T4). Different bone mineral parameters were measured by dual-energy X-ray at T4. Stepwise multiple regression analysis was performed to determine the effect of bone age, body mass and PA characteristics on measured bone mineral parameters at 18 years of age. *Results:* Total PA in puberty together with mean pubertal body mass predicted 35.5% of total body (TB) bone mineral density (BMD), 43.0% of TB less head (LH) bone mineral content (BMC) and 48.1% of BMC/height in individuals at 18 years of age. Vigorous PA and body mass in puberty predicted 43.2% of femoral neck (FN) BMD; bone age at T1, vigorous PA and body mass in puberty predicted 47.3% of FN BMC at 18 years of age. No associations between pubertal PA levels and lumbar spine bone mineral characteristics in individuals at 18 years of age were found. *Conclusions:* Physical activity in puberty has a significant impact on bone mineral characteristics in individuals at 18 years of age, with total PA being a significant predictor of TB BMD and TB LH BMC as well as BMC/height, whereas vigorous PA is a significant predictor of FN BMD and FN BMC.

## 1. Introduction

Osteoporosis is a major public health issue [1]. The peak bone mass achieved in youth is the strongest predictor of later life osteoporosis risk [2]. Genetic factors exert a strong influence on peak bone mass, but gene expression depends on hormone levels, nutrition and physical activity (PA) [3,4]. The pubertal years are a critical period regarding the accumulation of bone mass [5]. Factors that alter bone mass accumulation during this particular period of life may lead to suboptimal bone strength and higher fracture risk in late adulthood [2].

There is clear evidence for the positive effect of PA and exercise on bone mass and density during the late childhood and peri-pubertal years [6]. The positive relationships between PA and skeletal health can be explained by the bone’s mechanostat theory that describes the structural adaptation of bone tissues to their mechanical environment [7]. Mechanical loading tilts the balance between bone formation and resorption in favor of the former [8]. Physical activity has direct osteogenic effect via impact loading and via strains exerted on bone by muscle contractions [9]. Additionally, PA has indirect effect on bone by increasing muscle mass and hence the tensions generated on bone [9]. Adaptations in bone structure and strength related to PA are most often observed in pre- and peri-pubertal groups [10]. Exercise-induced osteogenic effect tends to plateau at end of puberty, suggesting that bones might be less sensitive to loading during this period of life [11].

It is known that PA decreases from childhood to adolescence [12,13] and tracks well throughout maturation period [14,15]. Therefore, understanding of the associations between PA and skeletal health in the critical period of maturation is of great importance.

Majority of studies investigating associations between objectively measured PA and bone mineral characteristics in children and adolescents are cross-sectional [16,17,18,19,20,21]. Bone mineral characteristics have been shown to be positively associated with PA intensities as well as total PA [18,20] and negatively associated with sedentary behavior [16,21] in numerous cross-sectional studies. Our previous cross-sectional studies have been in line with those findings, presenting associations between vigorous PA and femoral neck (FN) bone mineral density (BMD) in 11–13-year-old males [17,19].

Findings from longitudinal studies support the importance of PA for bone mineral accrual [22,23,24,25,26]. However, regarding PA, most of these studies have concentrated on the earlier stages of puberty, and the study periods have been relatively short. There are few longitudinal studies with longer than 5-year study period examining the effect of PA parameters on bone mineral outcome [27,28,29,30,31]. However, studies that addressed the outcome of adulthood bone mineral characteristics have measured PA indirectly via questionnaires [27,28] which can cause under- or over-reporting. A 12-year-long study demonstrated that high level of childhood PA was positively associated with bone strength in late adolescence even after drastic reduction in PA level during puberty [29]. Similarly, a negative association of sedentary time with bone geometry have been reported in a 4-year longitudinal study in adolescents [32].

Although some longitudinal studies examining the effect of PA on bone accrual in young subjects have been published, further evidence is needed to understand the relationships between PA and bone accrual in puberty and post-puberty. Therefore, the current study aimed to investigate whether PA at different intensities during puberty is related to bone mineral characteristics in individuals at 18 years of age.

## 2. Materials and Methods

### 2.1. Subjects and Study Design

The study conducted in 2017–2018 was a follow-up study of a longitudinal research project carried out between 2009–2013 [33,34]. At baseline, 313 boys participated in the study. Baseline data collection took place at 2010–2011. The invitation to participate in the current study was sent to all 217 subjects who had participated in 12-month and 24-month follow-up in 2011–2013. Of these, 104 boys agreed and participated in the follow-up. Valid accelerometer data were available in 88 subjects. Therefore, the final number of participants included into the analysis was 88.

The study was conducted following the Declaration of Helsinki, and the protocol was approved by the Research Ethics Committee of the University of Tartu (Estonia) (Consent No 260/T-19, 13 June 2016). All subjects and their parents were given a written description of the study and signed written informed consent was obtained from all subjects prior entering the study. If a participant was younger than 18.0 years, additional signed informed consent was retrieved from his parent.

Measurements of the current study included anthropometry, PA, sexual maturation and bone mineral characteristics. Four measurement sessions were performed. Anthropometry and PA were studied at baseline (T1), after one year (T2), after two years (T3) and after six years (T4). The mean age (years) of subjects at the study points were: 12.1 (range 10.6–13.4) at T1, 13.1 (11.6–14.0) at T2, 14.0 (12.5–15.3) at T3 and 18.0 (16.5–19.2) at T4 (Table 1). Sexual maturation and bone age assessment was performed at T1, T2 and T3. Bone mineral characteristics were studied at T4.

### 2.2. Anthropometry and Sexual Maturation

Body height (cm) was measured to the nearest 0.1 cm using Martin metal anthropometer according to the standard technique (GPM anthropological instruments, Zurich, Switzerland). Body mass (kg) was measured to the nearest 0.05 kg using medical electronic scale (A & D Instruments Ldt., Abington, UK) with the subject wearing light clothes. Body mass index (BMI; kg/m^2^) was calculated as body weight divided by square of body height.

Pubertal development was assessed by a self-report questionnaire of pubertal stages according to Tanner [35], which has been previously validated [36]. The subjects were given photographs, figures, and descriptions representing genitalia and pubic hair development stages and were asked to choose the one that most closely matched their own development. In the case of discrepancies between the two variables, Tanner stage of the subject was determined according to the self-estimation of genitalia development [36]. Bone age was determined by the method of Greulich and Pyle using an X-ray of the left hand and wrist [37].

### 2.3. Physical Activity

Physical activity was measured objectively at all four time-points of the study by ActiGraph accelerometer (model GT1M (at T1, T2 and T3) and model GT3X (at T4), ActiGraph LLC, Pensacola, FL, USA) designed to register vertical accelerations. All subjects were instructed to wear the accelerometer on the right hip for seven consecutive days during the wake-up time. For the analyses of accelerometer data, all night activity (24:00–6:00 h) and all sequences of 10 min or more of consecutive zero counts were excluded from each individual’s recording. At least two weekdays and one weekend day of recording with a minimum of 10 h/day was set as an inclusion criterion. The total PA was expressed as total number of counts divided by the registered time (counts/min). Time spent in PA with different intensity levels was calculated. The following cut-offs were used: sedentary time ≤ 100 counts/min, light PA 101–1999 counts/min, moderate PA 2000–3999 counts/min and vigorous PA ≥ 4000 counts/min [38]. The time spent in at least moderate intensity PA was calculated as moderate-to-vigorous PA (MVPA).

### 2.4. Bone Mineral Density

Dual-energy X-ray absorptiometry (Hologic QDR Series, Waltham, MA, USA) was used to measure BMD (g/cm^2^) and bone mineral content (BMC) (g) at the total body less head (TB LH), lumbar spine (LS) and FN. Bone area (BA) was assessed for the calculation of bone mineral apparent density (BMAD) (g/cm^3^), an estimate of volumetric bone density, according to the following formulas [39]:

TB BMAD = TB BMC/(TB BA^2^/height)

and

LS BMAD = LS BMC/BA^1.5^.

In addition, the expression of TB BMC for height (TB BMC/height) was calculated. The subjects were scanned in supine position wearing minimal clothing and medium scan mode was used for measurement. Coefficients of variation for bone mineral measurements were below 2%.

### 2.5. Statistical Analyses

Statistical analyses were performed using SPSS software version 20.0 for Windows (SPSS, Inc., Chicago, IL, USA). All variables were checked for normality of distribution before the analysis. Normally distributed continuous variables are described as a mean and +/− 1 SD, not normally distributed variables as a median and 25th and 75th percentile. Mean pubertal PA values including total PA, sedentary time, light PA, moderate PA, vigorous PA and MVPA were calculated using a formula:

Mean pubertal PA value = (PA value at T1 + PA value at T2 + PA value at T3)/3.

Using the same formula, mean pubertal body mass was calculated.

To determine the changes between different time-points of the study a paired t-test for normally distributed data and Mann-Whitney test for not normally distributed data were used. Spearman partial correlation analysis was performed to explore the relationships between mean pubertal PA variables and bone mineral data at T4, after controlling for baseline bone age and mean pubertal body mass.

Series of univariate models were performed to select the covariates for multiple regression model. Only variables that were statistically significant were included in the regression analyses. Stepwise multiple regression analysis was performed to determine the independent effect of bone age at T1, mean pubertal body mass and mean pubertal PA characteristics on measured bone mineral characteristics at T4. Statistical significance was set at *p* < 0.05.

## 3. Results

The anthropometrical and PA data of the subjects are presented in Table 1. The mean age of the boys at the beginning of the study was 12.1 years and the mean bone age 11.9 years. Regarding to their pubertal development the subjects at T1 were mainly at stage II (n = 33) and stage III (n = 45) by Tanner classification. Tanner stage, body mass, BMI and bone age increased significantly between time-points (*p* < 0.001) (Table 1). Sedentary time increased significantly during the puberty (*p* < 0.001) and time spent in light or moderate PA decreased (*p* < 0.001). Changes in vigorous PA were significant (*p* < 0.001): it increased from T1 to T2 and from T3 to T4, but decreased from T2 to T3. Nevertheless, when summed up, MVPA decreased at every time-point compared to the previous one (*p* < 0.001). Similarly, significant decrease over time was found in total PA (*p* < 0.001) (Table 1).

Bone mineral characteristics studied at T4 are presented in Table 2. Partial correlation coefficients between bone mineral characteristics in individuals at 18 years of age and mean pubertal PA levels where baseline bone age and mean pubertal body mass were entered as covariates, are presented in Table 3. Significant correlations between mean pubertal PA variables and FN BMD, TB LH BMC, FN BMC and BMC/height were found. FN BMD was positively correlated to mean vigorous PA (*r* = 0.303; *p* <0.005), mean MVPA (*r* = 0.260; *p* < 0.05) and total PA (*r* = 0.286; *p* < 0.005). Total body less head BMC was negatively correlated (*p* < 0.05) with mean sedentary time (r = −0.282) and positively with moderate PA (*r* = 0.263), vigorous PA (*r* = 0.307), MVPA (*r* = 0.326) and total PA (*r* = 0.380) (Table 3). Significant positive correlation was found between mean FN BMC and mean MVPA (*r* = 0.225; *p* < 0.05). BMC/height correlated negatively with mean sedentary time (*r* = −0.266; *p* < 0.05) and positively with total PA (*r* = 0.261; *p* < 0.05).

The results of stepwise multiple regression analysis, used to identify PA parameters in puberty that contribute most to bone mineral characteristics at T4, are presented in Table 4. Mean total PA in puberty together with mean body mass were associated with (*p* < 0.05) TB bone mineral characteristics whereas mean vigorous PA had an effect on FN BMD and BMC (Table 4). This analysis did not find any significant independent impact of PA on LS bone mineral characteristics.

## 4. Discussion

The findings of the present study indicate that total PA in puberty is an important predictor of TB BMD and TB LH BMC as well as BMC/height at 18 years of age, whereas vigorous PA in puberty is an important predictor of FN BMD and FN BMC in individuals at 18 years of age.

Our results from the stepwise multiple regression showed that total PA in puberty together with body mass in puberty is an important predictor of TB BMD at 18 years of age. This finding is supported by the study by Ivuškāns et al., in which changes in total PA were positively related to TB BMD increment in 11–13-year-old boys during a one-year study period [23]. Although Ivuškāns et al. argued that the effect of total PA on TB BMD was probably mediated by vigorous PA [23], our results from this study did not confirm this suggestion.

Similar to TB BMD, TB LH BMC in individuals at 18 years of age was also predicted by total PA measured in puberty. A longitudinal study of the relationship of PA measured by questionnaire to bone mineral accrual from adolescence to young adulthood in males and females aged 23 to 30 showed also a positive correlation between PA and TB BMC. Whereas active males had 8% greater TB BMC than their inactive counterparts, suggesting that the skeletal benefits of PA in adolescence are maintained into young adulthood [30]. Our results confirm the positive influence of pubertal PA on bone mass already at the age 18 years.

Results of our analyses showed that FN BMD in individuals at 18 years of age was positively correlated with mean pubertal vigorous PA, MVPA and total PA values, and FN BMC to MVPA. Vigorous PA in puberty was an important predictor of FN BMD and FN BMC also in stepwise regression analysis. The effect of vigorous PA on FN bone mass and density during puberty has been previously reported from cross-sectional [17,19,40] as well as from longitudinal [25,26] studies. The values of FN BMD and FN BMC showed the strongest association with PA parameters in comparison of different skeletal sites due to the highest responsiveness to physical stimulus [19]. There is evidence that effect of a short-term high-impact exercise intervention on hip BMC in early childhood is sustained through puberty [41]. Regarding habitual PA levels, children who accumulated the most MVPA in childhood had greater bone mass and better geometry at FN at the age of 17 years when compared to less active peers [29]. It has been found that active adolescent males had 9% higher adjusted FN BMC in adulthood than inactive adolescents [30]; however, as questionnaires were used to assess PA in the study, no conclusions about the individual effect of different intensity levels of PA, including vigorous PA, could be made. Although not directly comparable with the findings from our study due to different methods used to assess skeletal characteristics, Jackowsky et al. suggested that the skeletal benefits of PA in adolescence in FN are evident and maintained into adulthood [28]. Our findings about positive influence of vigorous PA on FN BMD and BMC support those from aforementioned studies.

Our results regarding PA levels in puberty were in line with those suggesting that PA decreases from childhood to adolescence [12,13,14,29]. We found a significant decrease in total PA throughout the study period accompanied with the increase in sedentary behavior and decrease in light and moderate PA. Vigorous PA increased from T1 to T2 and from T3 to T4; nevertheless, this did not result in an increase of MVPA. The mean duration of MVPA in our cohort reached the recommended amount of at least 60 min of MVPA a day [42] only at T1 (64.1 min/day) and T2 (59.9 min/day), after that MVPA decreased quite significantly being only 50.0 min/day at T4. The increase in sedentary behavior, together with an overall increase in vigorous PA and a decrease in total PA seen in our subjects during puberty, is in accordance with the conclusions drawn in the study of Metcalf et al. [14], suggesting that the decline in PA can be imputed to falls in light-intensity activity, rather than higher intensity activity.

In contrast to significant associations between total PA and BMD or BMC of TB and FN, the bone mineral characteristics describing more cortical bone, we found no significant impact of different pubertal PA parameters on LS BMD or LS BMC, the bone mineral characteristics describing more trabecular bone. One possible explanation for this might be that the patterns of increase in cortical and trabecular bone densities during growth differ considerably. A quantitative computer tomography study in healthy children suggested that weight-bearing activities are important determinants of cortical bone density while trabecular bones are influenced more by hormonal and/or metabolic factors associated with sexual development [43]. Similar differences between cortical and trabecular bone are were also seen in a study by Wang et al. concluding that Tanner stage I is a more sensitive period for PA in which to exert or exhibit its impact on appendicular skeletal development, whereas Tanner stage II or thereafter may be the most important time for the development of the BMD of LS or other axial skeletal sites [44]. Correspondingly, a study on relationships between PA and bone mass parameters in boys during pubertal growth spurt did not find a significant effect of PA on LS BMD [26].

Differences in loading responses of cortical and trabecular bone have been previously described in the findings of a study using the mouse tibial axial compression loading model to simultaneously explore cortical and trabecular bone adaptation to mechanical loading [45]. Dose response to loading magnitude within cortical bone was observed, with increasing load magnitude inducing increasing levels of cortical bone adaptation, whereas a dose response to load magnitude was not clearly evident within trabecular bone, with only the highest load being able to induce measurable adaptation to loading [45]. Relative importance of loading magnitude in osteogenic adaptation of trabecular bone seen in mouse model study is supported by the results in a large cohort of young men, revealing a positive association between high degree of mechanical loading, due to PA, and trabecular bone microstructure whereas the duration of PA was mainly related to cortical bone parameters [46]. Those lines of evidence about differences between bone mineral accrual in cortical and trabecular bone might explain why no significant associations between different pubertal PA parameters and LS bone mineral characteristics in individuals at 18 years of age were seen in our study.

A longitudinal study of long-term benefits of habitual PA during adolescence on adulthood LS bone characteristics of male subjects did not find significant difference between those who were physically active during adolescence compared to non-active counterparts. Subjects who were physically active during adolescence were defined as those who belonged to the highest quartile categorized according to the Z-scores of questionnaire-derived PA scores that were averaged over the entire adolescent period [31]. Nevertheless, in females who were active in adolescence, 3% greater trabecular content was seen compared to those who were inactive during adolescence [31]. These findings, together with our results, suggest that bone structural adaptation through childhood PA in males can be maintained into young adulthood, predominantly at the weight-bearing cortical bone sites.

One might argue that PA data from later stages of puberty and post-puberty should also have been measured and included into analyses when identifying PA parameters influencing bone mineral characteristics at 18 years of age. Nevertheless, a study on exercise-induced changes in bone mass and geometry suggests that bones might be less sensitive to loading from Tanner stages III to IV in male subjects [11]. Additionally, bone mineral characteristics at the age of 13 years might be influenced by increased PA level in the preceding years, whereas 3 years of continued high- or low-level activity up to age of 16 years did not yield any increased differences in bone size or bone mass [47]. We could assume that concerning the influence on PA on bone mineral outcome in individuals at 18 years of age, the PA from the age of 12 to 14 is of greater importance than PA in the following years.

The major strength of the current study is relatively long study period, including pubertal years that are known to be critical in regard to bone mineral accrual. Additionally, measuring PA objectively by using accelerometers with a short epoch adds strength to the study. The relatively small number of subjects and the absence of data on calcium intake should be considered as limitations in our study. In addition, our results are limited to males. A similar study in females would clarify whether the significant relationships between PA and bone mineral parameters from this study is relevant also for girls, as it is known that in pre- and early puberty boys’ bones are more sensitive to loading than girls’ bones, irrespective of muscle mass [18].

## 5. Conclusions

In conclusion, our study showed that PA in puberty has a significant impact on bone mineral characteristics in individuals at 18 years of age, and that total PA is an important predictor of TB BMD and TB LH BMC as well as BMC/height whereas the vigorous PA is a significant predictor of FN BMD and FN BMC. Our findings add to increasing evidence of the benefits of PA on bone mineral parameters, as they expand the knowledge of the particular role of pubertal years on that process.

## Figures and Tables

**Table 1:** Clinical characteristics and physical activity data of subjects (*n* = 88) at different timepoint of the study.

Variable	T1	T2	T3	T4
Clinical Characteristics				
Age (years)	12.1 (11.4–12.7)	13.1 (12.4–13.8) *	14.0 (13.4–14.7) *^,†^	18.0 (17.3–18.6)*^,†,‡^
Tanner	2.7 (2.04–3.37)	3.41 (2.64–4.18) *	4.13 (3.30–4.95) *^,†^	
[No. at stages (I/II/III/IV/V)]	1/33/45/9/0	0/8/43/30/7	0/2/18/33/35	
Height (m)	1.55 (1.46–1.63)	1.63 (1.54–1.72) *	1.69 (1.61–1.78) *^,†^	1.81 (1.75–1.88) *^,†,‡^
Body mass (kg)	47.15 (34.45–59.85)	54.00 (39.63–68.36) *	59.61 (45.90–73.32) *^,†^	73.86 (61.77–85.96) *^,†,‡^
BMI (kg/m^2^)	19.54 (15.58–23.49)	20.13 (16.05–24.21) *	20.60 (16.68–24.34) *^,†^	22.41 (19.14–25.69) *^,†,‡^
Bone age (years)	11.9 (10.73–13.0)	13.0 (11.8–14.2) *	13.9 (12.9–15.0) *^,†^	
Physical activity				
Sedentary time (min/day)	522.1 (485.9; 575.3)	539.1 (500.6; 585.7) *	562.1 (508.4; 627.5) *^,†^	623.5 (563.1; 680.1) ^†,‡^
Light PA (min/day)	220.5 (194.3; 244.25)	197.0 (166.0; 221.7) *	170.5 (138.8; 196.56) *^,†^	151.3 (133.7; 184.1) *^,†,‡^
Moderate PA (min/day)	45.8 (25.7; 59.4)	42.8 (32.4; 51.2) *	34.1 (27.1; 45.8) *^,†^	27.0 (17.8; 33.4) ^‡^
Vigorous PA (min/day)	15.1 (9.1;24.2)	16.5 (11.3; 23.8) *	13.7 (9.6; 27.8) *^,†^	25.3 (14.8; 35.1) *^,†,‡^
MVPA (min/day)	64.1 (46.25; 84.4)	59.9 (47.0; 75.4) *	52.0 (38.1; 67.7) *^,†^	50.0 (35.3; 69.7) *^,†,‡^
Total PA (counts/min)	434 (359; 573)	428 (346; 526) *	350 (283; 497) *^,†^	380 (303; 498) *^,†,‡^

* Significantly different (*p* < 0.05) from T1; † significantly different (*p* < 0.05) from T2; ‡ significantly different (*p* < 0.05) from T3. Median with 25th and 75th percentile for physical activity data and mean with +/− 1SD for anthropometry data are shown. BMI, body mass index; PA, physical activity; MVPA, moderate-to-vigorous physical activity.

**Table 2:** Bone mineral characteristics (mean with +/− 1SD) of the subjects (*n* = 88) at T4.

Variable	T4
TB BMD (g/cm^2^)	1.23 (1.14–1.32)
LS BMD (g/cm^2^)	1.06 (0.95–1.16
FN BMD (g/cm^2^)	1.01 (0.88–1.14)
TB LH BMC (g)	2323.04 (1965.03–2681.04)
LS BMC (g)	58.44 (49.10–67.78)
FN BMC (g)	5.09 (4.14–6.04)
TB BMAD (g/cm^3^)	0.095 (0.090–0.100)
LS BMAD (g/cm^3^)	0.143 (0.130–0.156)
BMC/height	1590.14 (1401.24–1779.05)

TB, total body; BMD, bone mineral density; LS, lumbar spine; FN, femoral neck; LH, less head; BMC, bone mineral content; BMAD, bone mineral apparent density.

**Table 3:** Partial correlation coefficients of bone mineral characteristics at T4 with mean ((T1 + T2 + T3)/3) physical activity variables.

	TB BMD	LS BMD	FN BMD	TB LH BMC	LS BMC	FN BMC	TB BMAD	LS BMAD	BMC/Height
	(g/cm^2^)	(g/cm^2^)	(g/cm^2^)	(g)	(g)	(g)	(g/cm^3^)	(g/cm^3^)	
Sedentary time (min/day)	−0.174	−0.072	−0.145	−0.282 *	−0.215	−0.091	0.075	0.084	−0.266 *
Light PA (min/day)	0.071	0.001	0.086	0.200	0.061	0.032	0.013	−0.050	0.090
Moderate PA (min/day)	0.199	0.099	0.149	0.263 *	0.118	0.166	0.106	0.046	0.206
Vigorous PA (min/day)	0.145	−0.021	0.303 *	0.307 *	0.138	0.226	0.066	−0.161	0.153
MVPA (min/day)	0.196	0.043	0.260 *	0.326 *	0.147	0.225 *	0.098	−0.069	0.205
Total PA (counts/min)	0.221	0.051	0.286 *	0.380 *	0.203	0.216	0.054	−0.109	0.261 *

* Statistically significant, *p* < 0.05. The correlations have been adjusted for baseline bone age and mean ((T1 + T 2+ T3)/3) body mass. TB, total body; BMD, bone mineral density; LS, lumbar spine; FN, femoral neck; LH, less head; BMC, bone mineral content; BMAD, bone mineral apparent density; PA, physical activity; MVPA, moderate-to-vigorous physical activity.

**Table 4:** The results of stepwise multiple regression analysis.

	R^2^ × 100	*F-*Ratio	Estimate	SE	*p* Value
TB BMD	35.5	21.489			<0.001
(Intercept)			0.963	0.046	<0.001
Body mass			0.004	0.001	<0.001
Total PA			<0.001	<0.001	0.038
LS BMD	18.1	17.955			<0.001
(Intercept)			0.559	0.110	<0.001
Bone age			0.039	0.009	<0.001
FN BMD	43.2	29.682			<0.001
(Intercept)			0.636	0.051	<0.001
Body mass			0.006	0.001	<0.001
Vigorous PA			0.003	0.001	0.003
TB LH BMC	43.0	29.426			<0.001
(Intercept)			1073.037	171.580	<0.001
Body mass			15.981	2.260	<0.001
Total PA			0.887	0.239	<0.001
LS BMC	10.0	8.749			0.004
(Intercept)			28.322	10.308	0.007
Bone age			2.553	0.863	0.004
FN BMC	47.2	22.960			<0.001
(Intercept)			0.423	0.893	0.637
Body mass			0.032	0.008	<0.001
Bone age			0.229	0.098	0.022
Vigorous PA			0.013	0.007	0.045
TB BMAD	-	-	-	-	-
LS BMAD	16.0	15.260			<0.001
(Intercept)			0.088	0.014	<0.001
Bone age			0.005	0.001	<0.001
BMC/height	48.1	36.093			<0.001
(Intercept)			934.242	88.275	<0.001
Body mass			9.699	1.163	<0.001
Total PA			0.306	0.123	0.015

Nine bone mineral characteristics entered as dependent variables and mean ((T1 + T2 + T3)/3) body mass, baseline bone age and mean ((T1 + T2 + T3)/3) physical activity variables (sedentary time, light PA, moderate PA, vigorous PA, MVPA and total PA) entered as independent variables. R2 × 100 value describes the percentage of variability of a bone mineral characteristic physical activity parameter, bone age and/or body mass will explain. SE, standard error; TB, total body; BMD, bone mineral density; LH, less head; PA, physical activity; LS, lumbar spine; FN, femoral neck; BMC, bone mineral content; BMAD, bone mineral apparent density.
